# Aldosterone Upregulates Transient Receptor Potential Melastatin 7 (TRPM7)*

**DOI:** 10.1074/jbc.M116.735175

**Published:** 2016-07-27

**Authors:** William C. Valinsky, Anna Jolly, Perrine Miquel, Rhian M. Touyz, Alvin Shrier

**Affiliations:** From the ‡ Department of Physiology, McGill University, Montreal, Quebec H3G 0B1, Canada and; the § Institute of Cardiovascular and Medical Sciences, University of Glasgow, BHF GCRC, 126 University Place, Glasgow G12 8TA, United Kingdom

**Keywords:** aldosterone, electrophysiology, mineralocorticoid receptor, plasma membrane, transient receptor potential channels (TRP channels), SGK1, α-kinase

## Abstract

Transient receptor potential melastatin 7 (TRPM7) is a ubiquitously expressed Mg^2+^-permeable ion channel fused to a C-terminal α-kinase domain. Recently, aldosterone was shown to increase intracellular Mg^2+^ levels and alter inflammatory signaling in TRPM7-expressing HEK293 cells. This study was undertaken to assess whether these effects were related to an aldosterone-mediated increase of TRPM7 current and/or plasma membrane localization. Using HEK293 cells stably expressing WT-TRPM7, we found that 18-h application of aldosterone significantly increased TRPM7 current and TRPM7 plasma membrane protein expression by 48% and 34%, respectively. The aldosterone-mediated increase of TRPM7 current was inhibited by eplerenone, a mineralocorticoid receptor (MR) blocker, and GSK-650394, an inhibitor of the serum- and glucocorticoid-regulated kinase 1 (SGK1). SGK1 blockade also prevented the aldosterone-induced increase of TRPM7 plasma membrane protein. It was further determined that K1648R-TRPM7, the phosphotransferase-inactive TRPM7 mutant, was unresponsive to aldosterone. Therefore, chronic aldosterone treatment increases the plasma membrane expression of TRPM7, which is associated with an increase of TRPM7 current. This process occurs via an MR-dependent, genomic signaling cascade involving SGK1 and a functioning TRPM7 α-kinase domain. We suggest that this mechanism may be of general relevance when interpreting the effects of aldosterone because the MR receptor is found in multiple tissues, and TRPM7 and SGK1 are ubiquitously expressed.

## Introduction

Aldosterone is a steroid hormone that regulates blood pressure by enhancing Na^+^ reabsorption (1) in the distal convoluted tubule (2), connecting tubule (3), and collecting duct (4, 5) of the nephron. These effects, as well as the homeostatic maintenance of Mg^2+^ and K^+^ (6, 7), are mediated by both genomic and non-genomic mechanisms. In the genomic cascade, aldosterone, through high-affinity interaction with the mineralocorticoid receptor (MR),^3^ increases the genomic expression of proteins associated with electrolyte regulation (8, 9). The best characterized protein, the epithelial Na^+^ channel (ENaC), is enhanced through increased mRNA synthesis (10, 11) and heightened biophysical function (12), leading to increased Na^+^ reabsorption from the urinary filtrate (11). Aldosterone also modulates ENaC through increased expression of the serum- and glucocorticoid-regulated kinase 1 (SGK1) (13, 14), which primarily enhances the plasma membrane expression of ENaC, leading to increased function (15–20).

The non-genomic actions of aldosterone have been divided into four pathways: MR-dependent (21), EGF receptor trans-activated (22), G-protein coupled receptor-dependent (23), and MR-independent (24, 25). Importantly, it has been shown that non-genomic activation of PKCα led to MR phosphorylation, and this was critical for the long-term genomic response to aldosterone (24). Thus, there may be cross-talk between the non-genomic and genomic signaling cascades.

Hyperaldosteronism, identified by elevated serum aldosterone levels (26), results in excessive Na^+^ reabsorption (27), K^+^ wasting (28), and Mg^2+^ wasting (6). In the kidney, the majority of Mg^2+^ reabsorption occurs in the thick ascending limb via paracellular transport, a process dependent on claudins 16 and 19 (29, 30). However, in the distal convoluted tubule, Mg^2+^ reabsorption occurs via transcellular pathways, implicating Mg^2+^-permeable ion channels (31, 32). Transient receptor potential melastatin 7 (TRPM7), a ubiquitously expressed Mg^2+^-permeable ion channel fused to a C-terminal α-kinase domain (33–37), has been identified as an aldosterone-responsive protein. It was reported that aldosterone, through non-genomic and genomic signaling cascades, increased intracellular Mg^2+^ levels and modulated inflammatory signaling via TRPM7 (38). However, it remains unclear whether these effects are associated with an actual change in TRPM7 function at the plasma membrane.

Using a combination of electrophysiological and biochemical techniques, we determined the effect of aldosterone on the TRPM7 channel. Our data demonstrate for the first time that aldosterone increases TRPM7 macroscopic current and that this is associated with elevated TRPM7 protein at the plasma membrane. Moreover, we show that this effect is mediated by the MR along with SGK1 and requires a functional TRPM7 α-kinase domain. Because SGK1 and TRPM7 are ubiquitously expressed, this may be a mechanism of general relevance associated with the activation of the MR by aldosterone in various tissues.

## Results

### 

#### 

##### Tetracycline Induction of WT-TRPM7

**FIGURE 1.**
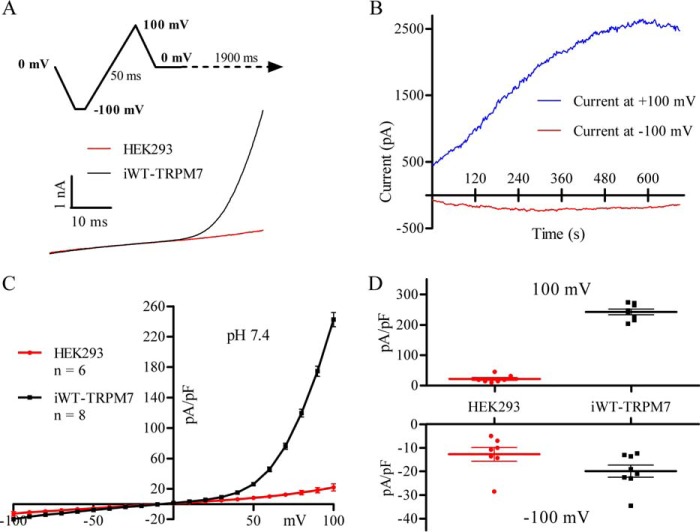
**Tetracycline induction of WT-hTRPM7 generates outwardly rectifying currents in HEK293 cells.** *A*, 50-ms ramp depolarization from −100 mV to +100 mV (*top panel*) was imposed to evoke TRPM7 current (*bottom panel*) in a non-transfected HEK293 cell (*red*) and an iWT-TRPM7 cell (*black*). *B*, growth of iWT-TRPM7 currents as a function of time following whole-cell configuration measured at +100 mV (*blue*) and −100 mV (*red*). Note that the current reaches a plateau level at about 7–10 min. *C*, I-V relationship obtained using the ramp protocol in non-transfected HEK293 cells (*n* = 6, *red*) and iWT-TRPM7 cells (*n* = 8, *black*). *D*, comparison of the current magnitudes between HEK293 cells and iWT-TRPM7 cells at +100 mV (*top panel*) and −100 mV (*bottom panel*). Data are expressed as mean ± S.E. in grouped analyses.

Whole-cell currents were evoked using a 50-ms voltage ramp from −100 mV to +100 mV (Fig. 1*A*). 18-h application of 1 μg/ml of tetracycline to HEK293 cells stably expressing WT-hTRPM7 in pcDNA4/TO (iWT-TRPM7) gave rise to a large macroscopic current that was not present in non-transfected HEK293 cells (Fig. 1, *A* and *C*). iWT-TRPM7 currents were outwardly rectifying-positive to 0 mV, reversed at 0 mV, and small and non-rectifying-negative to 0 mV. In accordance with prior reports (39, 40), iWT-TRPM7 currents were small after the initial patch rupture but, within 30 s, began to progressively increase until a peak was observed by 7–10 min (Fig. 1*B*). It can be seen in Fig. 1*D* that, compared with non-transfected HEK293 cells, currents were 1009% larger at +100 mV and 56% larger at −100 mV in iWT-TRPM7 cells.

##### Aldosterone Increases TRPM7 Current

**FIGURE 2.**
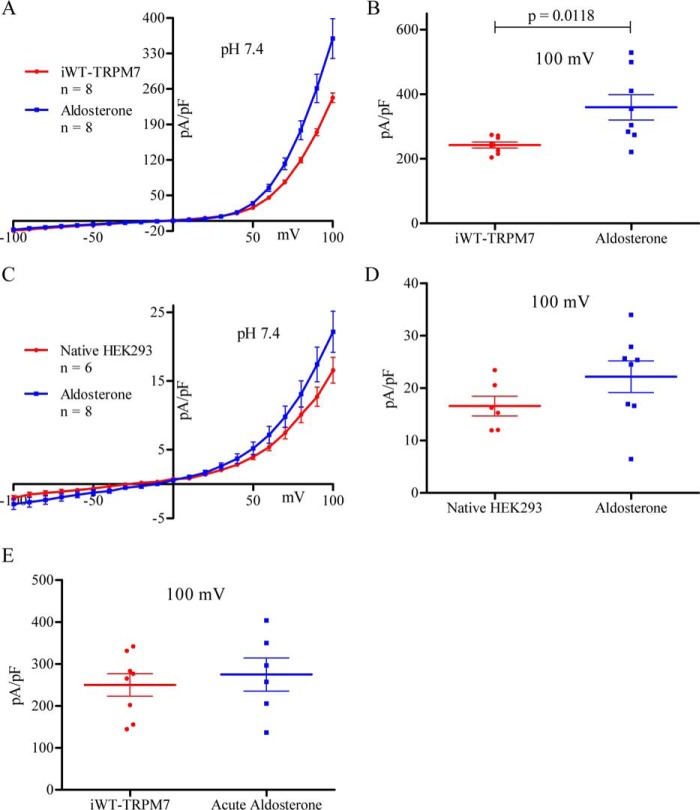
**Aldosterone treatment increases TRPM7 current.** *A*, I-V relationship of non-treated (*n* = 8, *red*) and 18-h aldosterone-treated (*n* = 8, *blue*) iWT-TRPM7 cells. *B*, current magnitude for the groups in *A* measured at +100 mV. *C*, I-V relationship of non-treated (*n* = 6, *red*) and 18-h aldosterone-treated (*n* = 8, *blue*) native HEK293 cells. *D*, current magnitude for the groups in *C* measured at +100 mV. *E*, current magnitude of iWT-TRPM7 cells before (*red*) and after (*blue*) aldosterone superfusion for 15–20 min (*n* = 6) measured at +100 mV. Statistical comparisons were performed using unpaired, two-way Student's *t* test. All data are expressed as mean ± S.E. Data were considered significant when *p* < 0.05.

TRPM7 has been implicated in the aldosterone-mediated increase of intracellular Mg^2+^ and altered inflammatory signaling (38). However, there was no evidence provided to support a direct effect of aldosterone on TRPM7 current. As shown in Fig. 2*A*, 18-h treatment of aldosterone (100 nm concurrently with tetracycline) increased the outward current limb of the iWT-TRPM7 I-V relationship so that a statistically significantly increase was observed at +100 mV (243 ± 9 pA/pF to 360 ± 39 pA/pF) (Fig. 2*B*). This significant increase was due to an effect on recombinant WT-TRPM7, as native HEK293 currents were not significantly increased by aldosterone treatment, although the mean current density at +100 mV was slightly larger (22 ± 3 pA/pF *versus* 17 ± 2 pA/pF, respectively) (Fig. 2, *C* and *D*). Furthermore, although there are reports of low-levels of TRPM7 expression in HEK293 cells (38), it is not entirely clear what fraction of the endogenous macroscopic current TRPM7 constitutes, as the HEK293 I-V relationship is not unique to TRPM7 (41). Thus, it is uncertain what fraction of the HEK293 macroscopic current would be aldosterone-responsive.

To assess whether aldosterone could modulate TRPM7 current through rapid, non-genomic pathways, iWT-TRPM7 cells were assessed under acute aldosterone treatment. No changes in peak current were observed when iWT-TRPM7 cells were superfused with 100 nm aldosterone and recorded for 15–20 min (Fig. 2*E*). This duration was chosen in accordance with prior studies examining non-genomic aldosterone signaling (21–25, 42–47).

##### MR and SGK1 Mediate the Effect of Aldosterone on TRPM7

**FIGURE 3.**
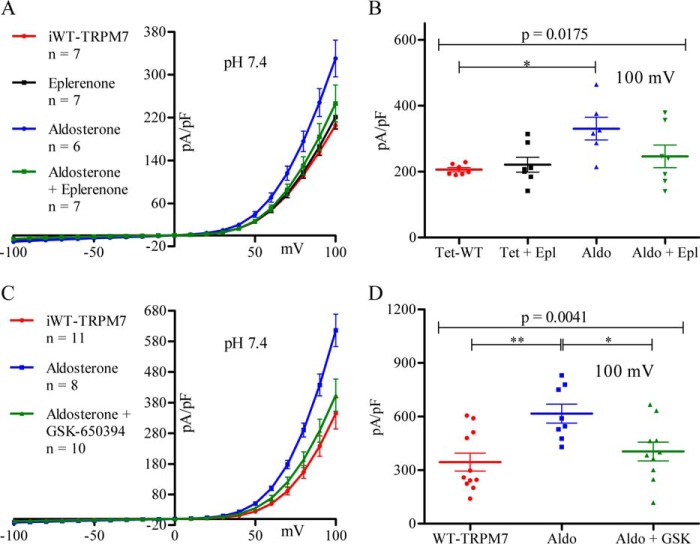
**Aldosterone induces TRPM7 macroscopic current via the MR and SGK1.** *A*, I-V relationship of iWT-TRPM7 cells treated with (*n* = 8, *black*) or without (*n* = 8, *red*) eplerenone as well as iTRPM7 cells treated with aldosterone (*n* = 6, *blue*) or with aldosterone + eplerenone (*n* = 7, *green*). *B*, current magnitude for the groups examined in *A* measured at +100 mV. C, I-V relationship comparing non-treated (*n* = 11, *red*), aldosterone-treated (*n* = 8, *blue*), and aldosterone + GSK-650394 (*n* = 10, *green*) iWT-TRPM7 cells. *D*, current magnitude for the groups compared in *C* measured at +100 mV. Statistical comparisons were performed using one-way ANOVA with Bonferroni post-hoc tests. All data are expressed as mean ± S.E. Data were considered significant when *p* < 0.05. *, *p* ≤ 0.05; **, *p* ≤ 0.01.

To test the involvement of the MR, we employed the MR antagonist eplerenone (48) at an IC_50_ concentration of 360 nm (49). Fig. 3*A* shows that eplerenone diminished the aldosterone-induced increase of iWT-TRPM7 current. At +100 mV, peak currents were 206 ± 6 pA/pF for iWT-TRPM7 cells, 330 ± 34 pA/pF for aldosterone-treated iWT-TRPM7 cells, and 246 ± 34 pA/pF for aldosterone/eplerenone-treated iWT-TRPM7 cells (Fig. 3*B*). The difference between the peak outward currents of non-treated and aldosterone/eplerenone-treated iWT-TRPM7 cells was not statistically significant, indicating that MR blockade prevented the aldosterone-mediated increase in TRPM7 current.

SGK1 is a secondary mediator of the aldosterone/MR genomic signaling cascade (50) known to increase the expression (10, 11), surface accumulation (15, 51), and function (12) of ENaC, resulting in increased Na^+^ reabsorption in the distal nephron (13). We tested whether SGK1 function was necessary for the aldosterone effect observed in TRPM7-expressing HEK293 cells using 6 μm GSK-650394, a compound that inhibits the enzymatic activity of SGK1 (52). Concurrent treatment of iWT-TRPM7 cells with aldosterone and GSK-650394 resulted in an I-V relationship essentially identical in magnitude to non-treated iWT-TRPM7 cells (Fig. 3*C*). At +100 mV, iWT-TRPM7 current was 616 ± 51 pA/pF for aldosterone-treated cells, 404 ± 52 pA/pF for aldosterone/GSK650394-treated cells, and 345 ± 51 pA/pF for non-treated cells (Fig. 3*D*). The difference in peak current between non-treated and aldosterone/GSK-650394-treated iWT-TRPM7 cells was not statistically significant, indicating that SGK1 blockade also prevented the aldosterone-mediated increase in TRPM7 current.

**FIGURE 4.**
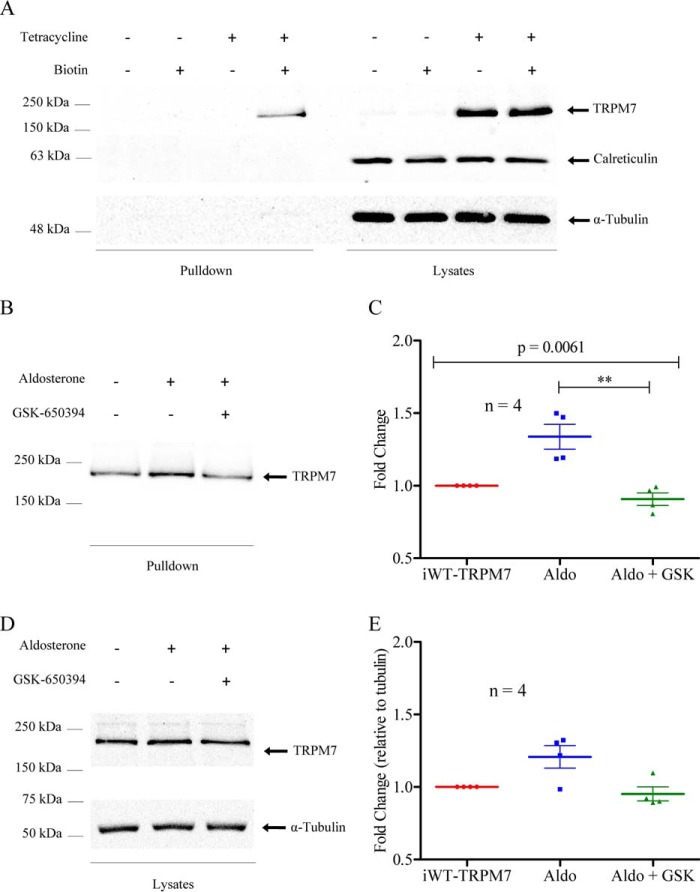
**Aldosterone increases WT-TRPM7 plasma membrane protein expression.** *A*, representative Western blot showing TRPM7 biotinylated (*left panel*) and total lysate (*right panel*) samples. Calreticulin is presented as a control for biotinylation plasma membrane specificity. α-Tubulin is presented as a loading control. *B*, Western blot showing plasma membrane expression of TRPM7 in non-treated, aldosterone-treated, and aldosterone + GSK-650394-treated iWT-TRPM7 cells. *C*, quantification (*n* = 4) of plasma membrane expression. *Aldo*, aldosterone. *D*, Western blot showing TRPM7 lysate levels in non-treated, aldosterone-treated, and aldosterone + GSK-650394-treated iWT-TRPM7 cells. *E*, quantification (*n* = 4) of protein lysate levels. Lysate data were normalized to α-tubulin. Statistical comparisons were performed using Kruskal-Wallis with Dunn's post-hoc tests. Data are expressed as mean ± S.E. in grouped analyses. Data were considered significant when *p* < 0.05. **, *p* ≤ 0.01.

A common mechanism by which SGK1 increases channel activity is via increased plasma membrane expression, a result observed for ENaC (15), renal outer medullary potassium channel 1 (ROMK1) (53), ClC-Ka (54), and transient receptor potential vanilloid 5 (TRPV5) (55). To assess whether SGK1 increased the plasma membrane expression of TRPM7, biotinylation protocols were utilized. Fig. 4*A* shows a sample blot of biotin pulldowns (*left panel*), indicative of plasma membrane proteins, and whole-cell lysates (*right panel*), indicative of total protein, for HEK293 cells expressing WT-hTRPM7. A single band of ∼220 kDa, the expected size of TRPM7 (34), was detected in the iWT-TRPM7 biotinylated pulldowns. A stronger band of the same size was also detected in the iWT-TRPM7 lysates. To rule out potential plasma membrane non-specificity of the biotinylated products, we blotted for calreticulin, a protein localized to the endoplasmic reticulum (56). Fig. 4*A* further shows that calreticulin was only present in the whole-cell lysates and not in the pulldown products. When treated with aldosterone, iWT-TRPM7 plasma membrane expression increased by 34% (*p* = 0.0291; Fig. 4, *B* and *C*), indicating that aldosterone significantly increased plasma membrane TRPM7. This increase was prevented by co-application of GSK-650394 (Fig. 4, *B* and *C*), supporting the patch clamp data above. Changes in iWT-TRPM7 whole-cell lysate levels (normalized to α-tubulin) were also observed with aldosterone application (increase of 21%); however, these results were not statistically significant (Fig. 4, *D* and *E*).

##### Aldosterone Does Not Change TRPM7 Voltage Dependence of Activation

**FIGURE 5.**
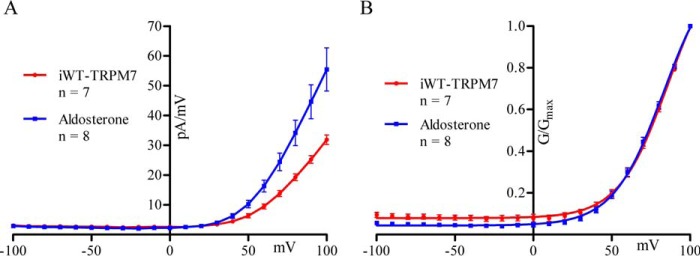
**TRPM7 voltage dependence of activation is not changed by aldosterone.** *A*, G-V relationship of non-treated (*n* = 7, *red*) and aldosterone-treated (*n* = 8, *blue*) iWT-TRPM7 cells. *B*, normalized conductance (G/G_max_)-voltage relationship of *A* fitted to the Boltzmann equation. Data are expressed as mean ± S.E. for the I-V plot and mean ± S.D. for the Boltzmann function.

G-V relationships for non-treated and aldosterone-treated iWT-TRPM7 cells are shown in Fig. 5*A* and normalized in Fig. 5*B*. The two normalized G-V relationships in Fig. 5*B* were fit with the Boltzmann function. The V_50_ values (the voltage at which the G/G_max_ is 0.5) (86 ± 2 mV for non-treated and 83 ± 2 mV for aldosterone-treated) and slopes (16 for non-treated and 16 for aldosterone-treated) were highly similar, indicating that the increase of TRPM7 current attributed to aldosterone treatment was not associated with changes in TRPM7 voltage dependence of activation.

##### Aldosterone Requires a Functional TRPM7 α-Kinase Domain to Enhance TRPM7 Current

**FIGURE 6.**
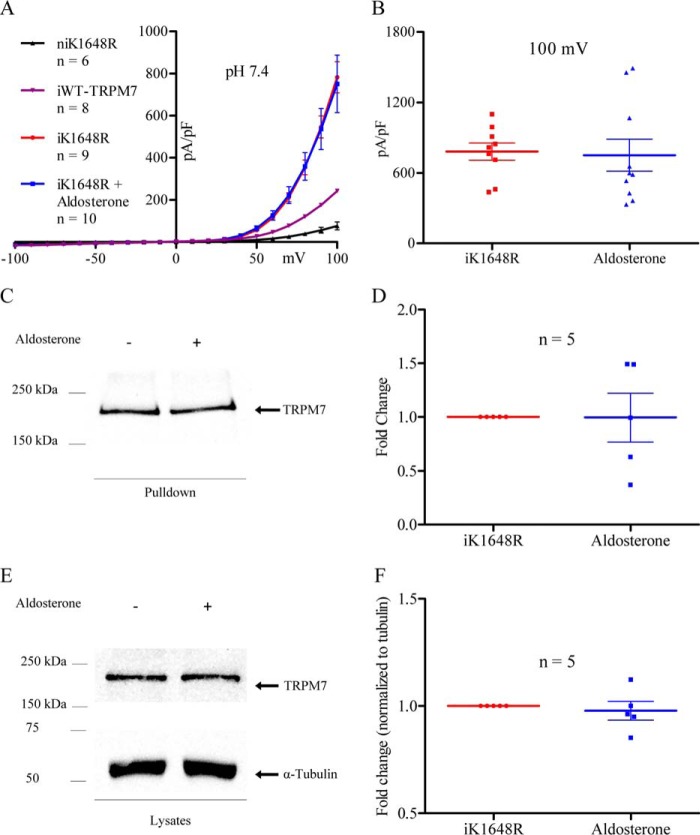
**Aldosterone-stimulated increase of TRPM7 macroscopic current and plasma membrane expression requires a functional TRPM7 α-kinase.** *A*, I-V relationship of non-treated iWT-TRPM7 cells (*n* = 8, *purple*), non-induced K1648R-TRPM7 cells (*n* = 6, *black*), non-treated iK1648R-TRPM7 cells (*n* = 9, *red*), and aldosterone-treated (*n* = 10, *blue*) iK1648R-TRPM7 cells. *B*, comparison of current magnitude between the groups in *A* measured at +100 mV. *C*, Western blot showing plasma membrane expression of TRPM7 in non-treated and aldosterone-treated iK1648R-TRPM7 cells. *D*, quantification (*n* = 5) of plasma membrane expression. *E*, Western blot showing lysate levels of non-treated and aldosterone-treated iK1648R-TRPM7 cells. *F*, quantification (*n* = 5) of protein lysate levels. Lysate data were normalized to α-tubulin. Patch clamp data were statistically compared using unpaired, two-way Student's t-tests. Western blotting data were statistically compared by one-sample Wilcoxon signed-rank test. Data are expressed as mean ± S.E. Data were considered significant when *p* < 0.05.

To determine whether the TRPM7 α-kinase was required for the aldosterone-induced increase of TRPM7 plasma membrane expression and whole-cell current, we studied HEK293 cells stably expressing a TRPM7 α-kinase phosphotransferase-inactive mutant, K1648R-TRPM7. When treated with aldosterone, no significant changes in induced K1648R-TRPM7 (iK1648R-TRPM7) currents (Fig. 6, *A* and *B*), plasma membrane expression (Fig. 6, *C* and *D*), or total protein (Fig. 6, *E* and *F*) were observed. Thus, a functional TRPM7 α-kinase is necessary for aldosterone to increase TRPM7 plasma membrane expression and macroscopic current.

## Discussion

In this study, we demonstrate that aldosterone increases the plasma membrane expression of TRPM7. This effect is mediated by the MR and is SGK1-dependent. Furthermore, a functional TRPM7 α-kinase is required for the aldosterone-stimulated increase of TRPM7 plasma membrane expression and macroscopic current.

The results of this study support the notion that aldosterone signaling via TRPM7 is mediated through genomic and kinase-dependent mechanisms (38). Moreover, our results suggest that these previously observed effects were associated with changes in TRPM7 plasma membrane expression and whole-cell current, indicating that the effect of aldosterone involves functional TRPM7 channels. However, our results also suggest that the previously observed acute increase of intracellular Mg^2+^ in aldosterone-treated iWT-TRPM7 cells was not caused by increased Mg^2+^ transport via TRPM7, as there were no acute aldosterone-induced increases of iWT-TRPM7 macroscopic current. It is possible that the increased intracellular Mg^2+^ observed in the earlier study was due to the activation of another Mg^2+^ transporter, such as the ubiquitously expressed magnesium transporter 1 (MagT1) (57, 58), which was identified as an important protein in Mg^2+^ uptake (58, 59).

The results of this study also implicate SGK1, which is expressed in virtually all tissues (60) and has been shown to be genomically upregulated by aldosterone (61). SGK1, in turn, regulates a variety of ion channels (15, 53–55, 62–65). The most common mechanism by which SGK1 regulates ion channels is through increased ion channel plasma membrane abundance, a process documented for the renal ion channels ENaC (15), ROMK1 (53, 62), ClC-Ka (54), and TRPV5 (55). In this study, we found that aldosterone induced similar increases in TRPM7 current (48%) and TRPM7 plasma membrane expression (34%). The difference between these values is most likely attributable to the semiquantitative nature of the biotinylation assay. Moreover, the voltage dependence of TRPM7 activation did not change in response to aldosterone treatment. Therefore, we suggest that SGK1 increases TRPM7 plasma membrane expression, resulting in increases in TRPM7 macroscopic current.

The role of the TRPM7 α-kinase in channel function has been assessed previously. Point mutations within the TRPM7 α-kinase do not affect TRPM7 current magnitude but alter the inhibitory capacity of Mg^2+^ nucleotides (66). With regards to aldosterone, our primary conclusion based on the kinase-mutant data is that a loss of phosphotransferase activity abolishes the aldosterone response, suggesting that SGK1 regulation of TRPM7 occurs via the α-kinase.

The mechanisms behind Mg^2+^ wasting in hyperaldosteronism remain uncertain. The results of this study demonstrate that aldosterone increases the plasma membrane expression of TRPM7, resulting in increased macroscopic current. However, Mg^2+^ wasting is difficult to assess in an isolated context, as 14 Mg^2+^ channels or transporters have been identified in renal distal convoluted tubular cells (32, 57, 67–74), where aldosterone would have a biological function (3, 75).

The relationship between aldosterone, SGK1, and TRPM7 is of general significance, as SGK1 (60) and TRPM7 (33) are ubiquitously expressed. Therefore, TRPM7 may be upregulated in any cell stimulated by aldosterone. For example, in the acidic distal tubule, where aldosterone and SGK1 have been implicated previously (15, 53–55, 62), apically located TRPM7 would function as an Na^+^ channel (40), increasing intracellular Na^+^ transport and, therefore, Na^+^ reabsorption. However, in vascular cells, which synthesize aldosterone (76) and also express the required aldosterone genomic signaling moieties (77–79), TRPM7 would function as a divalent cation channel, increasing the intracellular transport of Mg^2+^ and Ca^2+^ and participating in the inflammatory cascade (38). Thus, there may be different functional consequences of TRPM7 upregulation by aldosterone. Furthermore, the potential widespread nature of the aldosterone/TRPM7 response should be considered in future studies investigating aldosterone pathologies.

## Experimental Procedures

### 

#### 

##### Tissue Culture and TRPM7 Induction

HEK293 cells were grown in DMEM (low-glucose, Gibco) supplemented with 10% FBS (Wisent Bioproducts), 100 units/ml penicillin, and 100 μg/ml streptomycin (Gibco). 400 μg/ml zeocin (Invitrogen) and 5 μg/ml blasticidin (Invitrogen) were further added to maintain stable expression of TRPM7 constructs. Cells were cultured at 37 °C in 5% CO_2_. Cell medium was changed every 3–4 days, and cells were passaged every 4–5 days via trypsinization.

HEK293 cells were stably transfected with HA-tagged WT hTRPM7 or phosphotransferase-inactive K1648R hTRPM7 in pcDNA4/TO vectors (38, 66). This expression system has been used in prior electrophysiological studies of TRPM7 (33, 35, 40, 80). TRPM7 expression was induced by the addition of 1 μg/ml tetracycline (Sigma) 18 h prior to experimentation, as described previously (38, 66). In experiments examining aldosterone, cells were concurrently induced with tetracycline (18 h) and treated (18 h) with 100 nm aldosterone (Sigma) (0.004% ethanol vehicle) unless indicated otherwise. In experiments utilizing antagonists, cells were also treated (18 h) with 360 nm eplerenone (0.1% DMSO vehicle) (Tocris) or 6 μm GSK-650394 (0.1% DMSO vehicle) (Tocris). Cell induction and treatments were performed on 60–90% confluent HEK293 cells grown in 60- or 100 mm-diameter Petri dishes.

##### Electrophysiology

Cells were plated in the perfusion chamber of an inverted microscope (Zeiss Axiovert S100TV) and perfused at a rate of 1–2 ml/min with Tyrode solution (145 mm NaCl, 5.4 mm KCl, 1.8 mm CaCl_2_, 1 mm MgCl_2_, and 5 mm HEPES (pH to 7.4 with NaOH, 285 mosmol)). Patch pipettes were fabricated using borosilicate glass capillaries (Warner Instruments, Hamden, CT) and a microprocessor-controlled, multistage micropipette puller (P97, Sutter Instruments). Pipettes with resistances of 1.5–3 megohms were backfilled with pipette solution (130 mm CsCl, 10 mm NaCl, 10 mm Cs_4_1,2-bis(*o*-aminophenoxy)ethane-*N*,*N*,*N′*,*N′*-tetraacetic acid, and 10 mm HEPES (pH to 7.2 with CsOH, 285 mosmol)). Patch clamp experiments were performed at room temperature (∼21 °C).

Cells were recorded in the whole-cell configuration, as perforated patch is not optimal for TRPM7 current growth (81). Whole-cell experiments were conducted using an Axopatch 200B amplifier coupled with a CV 203BU headstage. Data were obtained using pClamp 10.4 and a Digidata 1440A digitizer (Axon Instruments). Data were sampled at 20 kHz and filtered at 2 kHz. To limit cellular toxicity and variation in tetracycline-induced currents, cells were recorded 18–24 h after induction (35). *n* refers to the number of cells recorded, each from a separate dish. The total *n* for each experimental condition was generated from a minimum of three separate inductions.

Prior to the formation of a gigaohm seal, currents were corrected for pipette (fast) capacitance. Upon formation of the whole cell-configuration, cell capacitance was determined using a 30-ms, 10-mV depolarizing pulse from a holding potential of −80 mV at 2 Hz. Currents were corrected for whole-cell capacitance and series resistance compensated to 80%. All recorded cells had access resistances below 10 megohms.

The liquid junction potential (LJP) between the Tyrode solution and the pipette solution (2 mV) was compensated offline using the formula V_membrane_ = V_Pipette_ − V_LJP_ (82). I-V relationships were obtained using a 50-ms ramp protocol from −100 to +100 mV, from a holding potential of 0 mV, at 0.5 Hz. The protocol was applied for 700 s and repeated when necessary.

Currents in the I-V relationships are presented as current density (picoampere/picofarad), where picofarad was determined by cellular capacitance. Where appropriate, G-V relationships are presented. Conductance was calculated by dividing the recorded current (picoampere) by the driving force (V_driving_ = V_test_ − V_reversal_). To fit G-V relationships to a Boltzmann function, plots were normalized to peak conductance and are presented as G/G_max_. In cells expressing TRPM7, current magnitude increased over time following patch rupture for ∼7–10 min until a maximum current was observed (Fig. 1*B*). I-V relationships are presented as maximal currents.

##### Biotinylation

Upon removal from the incubator, cells were maintained at 4 °C and washed on ice with ice-cold PBS (140 mm NaCl, 3 mm KCl, 10 mm Na_2_HPO_4_, and 2 mm KH_2_PO_4_) containing 1 mm MgCl_2_ and 0.1 mm CaCl_2_ (pH 8.0) (PBS-CM). Cells were incubated with 0.5 mg/ml EZ-Link^TM^ sulfo-NHS-SS-biotin (Thermo Scientific) in PBS-CM for 30 min on ice. Cells were washed and then incubated with quenching buffer (50 mm glycine in PBS-CM) for 7 min on ice. This step was repeated a second time. Cells were washed two more times with PBS-CM and then harvested and lysed in radioimmunoprecipitation assay buffer (150 mm NaCl, 20 mm Tris-HCl (pH 8), 1% Triton X-100, 0.1% SDS, and 0.5% sodium deoxycholate). A small fraction of the cell lysates was removed for use in Western (lysate) blotting, and the remainder was incubated with 100 μl of 50% Neutr-Avidin®-agarose slurry (Thermo Scientific) in RIPA buffer with a protease inhibitor mixture (Roche Applied Science) overnight at 4 °C. The biotinylated protein complexes were washed four times in RIPA buffer and eluted with 2× Laemmli loading buffer (100 mm Tris-HCl (pH 6.8), 10% glycerol, 10% SDS, and 10% β-mercaptoethanol) for 45 min at room temperature. Samples were then used in Western blotting.

##### Western Blotting

Samples were loaded and separated by 6% SDS-polyacrylamide gel electrophoresis, and protein bands were subsequently transferred onto nitrocellulose membranes (Bio-Rad). Membranes were blocked with a 5% milk solution and then incubated overnight with antibodies at 4 °C. Immunoblots were incubated with secondary antibodies and visualized using Amersham Biosciences ECL Western blotting detection reagent (GE Healthcare Life Sciences). α-Tubulin was used as a loading control. After observing no intracellular calreticulin or α-tubulin with biotinylation (elution) blots (Fig. 4*A*), all subsequent elution blots are depicted without controls, as presented previously (83). Antibodies used were as follows: anti-TRPM7 (NeuroMab, clone N74/25, monoclonal), anti-α-tubulin (Sigma, monoclonal), anti-calreticulin (Stressgen, polyclonal), peroxidase-conjugated AffiniPure goat anti-rabbit (Jackson ImmunoResearch Laboratories, polyclonal), and peroxidase-conjugated AffiniPure goat anti-mouse (Jackson ImmunoResearch Laboratories, polyclonal).

##### Statistical Analysis

All statistical analyses were performed in GraphPad Prism 5.0. Data are expressed as mean ± S.E. unless indicated otherwise. Data from the Boltzmann function are presented as mean ± S.D. To evaluate significance between two groups, unpaired, two-way Student's *t* tests were used unless otherwise indicated. To evaluate statistical significance between three groups, one-way ANOVA with Bonferroni post-hoc tests were used. To evaluate statistical significance for normalized protein quantification between two groups, a one-sample Wilcoxon signed-rank test was used. To evaluate statistical significance for normalized protein quantification between three groups, Kruskal-Wallis with Dunn's post-hoc tests were used. In accordance with prior studies studying aldosterone (21, 84–88) and SGK1 (89), whole-cell lysates data were normalized to α-tubulin. Furthermore, in this study, α-tubulin levels were unaffected by aldosterone treatment, as relative to control, α-tubulin quantification was 0.88 for aldosterone and 0.89 for aldosterone + GSK-650394 in iWT-TRPM7 cells. Data were considered significant when *p* < 0.05. Precise *p* values are depicted for ANOVA, Kruskal-Wallis test, *t* test, and rank test results. In post-hoc tests, statistical significance is indicated as follows: *, *p* ≤ 0.05; **, *p* ≤ 0.01; ***, *p* ≤ 0.001; ****, *p* ≤ 0.0001.

## Author Contributions

All experiments were performed in the laboratory of A. S. W. C. V. designed, acquired, analyzed, and interpreted all patch clamp results and wrote the manuscript. A. J. designed, acquired, analyzed, and interpreted all blotting experiments and helped write the manuscript. P. M. helped design and perform preliminary patch clamp experiments. R. M. T. and A. S. funded and supervised the study, aided with data interpretation, and helped write the manuscript. All authors have read and approved the final submission.

## Footnotes

3 The abbreviations used are:

MR: mineralocorticoid receptor
ENaC: epithelial sodium channel
pF: picofarad
I-V: current-voltage
G-V: conductance-voltage
LJP: liquid junction potential
RIPA: radioimmune precipitation assay
ANOVA: analysis of variance.
